# Heat shock transcription factor 1 acts as an endogenous protective mechanism in mechanically stretched alveolar epithelial cells

**DOI:** 10.1016/j.cstres.2026.100196

**Published:** 2026-07-10

**Authors:** Jinqiu Ding, Xinyi Tang, Haoyue Xue, Zuxin Zhou, Haoran Chen, Yao Yan, Yongpeng Xie

**Affiliations:** 1 Department of Emergency Medicine, The Affiliated Lianyungang Hospital of Xuzhou Medical University, The Lianyungang Clinical College of Nanjing Medical University, The First Affiliated Hospital of Kangda College of Nanjing Medical University, The First People's Hospital of Lianyungang, Lianyungang, Jiangsu 222000, China; 2 Department of Intensive Care Medicine, Affiliated Hospital of Nantong University, Nantong, Jiangsu 226000, China

**Keywords:** Alveolar epithelial cells, Heat shock transcription factor 1, Heat shock proteins, Mechanical stretch, Ventilator-induced lung injury

## Abstract

Mechanical ventilation is a key respiratory support measure for critically ill patients. During improper ventilation, continuous exposure of alveolar epithelial cells (AECs) to abnormal mechanical environment can lead to ventilator-induced lung injury (VILI). Heat shock transcription factor 1 (HSF1) is a stress-responsive transcriptional regulator that orchestrates cytoprotective heat shock protein (HSP) expression in response to diverse stresses, including thermal and oxidative stress, but its role in stretch-induced AEC injury remains unclear. In this study, A549 cells were subjected to biaxial cyclic stretch to model mechanical stress associated with VILI in vitro. Consistent with the established role of YAP as a mechanotransduction regulator activated by cyclic stretch, we observed YAP signaling activation in our biaxial stretch system. Transcriptomic analysis revealed that mechanical stretch markedly induced the expression of HSP genes, and subsequent validation confirmed that stretch activated HSF1 and increased HSF1-dependent HSP expression. Pharmacological inhibition of HSF1 with DTHIB or CRISPR-Cas9-mediated HSF1 knockout attenuated stretch-induced HSP expression, while exacerbating ROS accumulation, cell death, and IL-6 production. Moreover, oxidative-stress-associated HSF1 targets were significantly enriched among stretch-induced genes, antioxidant treatment partially suppressed stretch-induced HSF1 activation, and HSF1 knockout increased oxidative stress after stretch. These findings indicate that HSF1 functions as an endogenous cytoprotective feedback mechanism in mechanically stretched AECs by inducing HSP expression and limiting stretch-induced oxidative damage and inflammatory injury. Activation of the HSF1-HSP axis may therefore represent a potential strategy for mitigating epithelial injury during VILI.

## Background

Mechanical ventilation (MV) is a cornerstone of critical care for patients with acute respiratory failure; however, its inappropriate application can precipitate ventilator-induced lung injury (VILI),^1^ a syndrome characterized by alveolar overdistension, inflammatory amplification, and epithelial–endothelial barrier disruption.^2^ The classical pathogenesis of VILI includes barotrauma, volutrauma, atelectrauma, and biotrauma.^3^ Despite the implementation of protective ventilation strategies such as low tidal volume, restricted plateau pressure, and titrated positive end-expiratory pressure, VILI remains an important contributor to morbidity and mortality in critically ill patients.^4^ The pathogenesis of VILI has not been fully clarified, and effective treatment measures remain limited. Therefore, it is of great significance to further explore the molecular mechanisms underlying VILI and identify potential therapeutic targets.

Alveolar epithelial cells (AECs) are strategically positioned at the air–tissue interface and are subjected to cyclic tensile forces during MV.^5^ AEC damage plays an important role in the development of VILI. Through mechanosensation and mechanotransduction, AECs convert extracellular mechanical signals into intracellular biochemical signals that regulate cellular physiological functions.^6^ Yes-associated protein (YAP) is a key mechanotransduction regulator that responds to mechanical force and acts as a transcriptional coactivator to regulate cell proliferation, regeneration, differentiation, and inflammatory responses.^7^, ^8^, ^9^ Previous studies have shown that mechanical stretch can regulate YAP phosphorylation and nuclear localization in epithelial cells, thereby modulating YAP-dependent transcriptional programs.^8^, ^9^ Therefore, YAP activation provides a useful readout for evaluating the biological response of AECs to cyclic stretch.

The heat shock response (HSR) is an evolutionarily conserved cytoprotective program that preserves proteostasis under diverse stress conditions, including heat and oxidative stress.^10^, ^11^, ^12^, ^13^ Heat shock proteins (HSPs), particularly members of the HSP70 family, function as ATP-dependent molecular chaperones that assist protein folding, refold stress-denatured proteins, and cooperate with protein quality-control pathways to prevent proteotoxic damage.^14^ According to standardized nomenclature guidelines, HSPs are commonly classified into several families, including HSP110 family, HSP90 family, HSP70 family (HSPA), HSP60/HSP10 chaperonin families, HSP40 family, and small heat shock protein family (HSPB).^15^ Central to the classical HSR is heat shock transcription factor 1 (HSF1), a stress-responsive transcription factor that undergoes activation, nuclear translocation, and binding to heat shock elements in target gene promoters to induce a subset of stress-inducible HSPs.^11^, ^12^ Beyond canonical proteostasis, HSF1–HSP signaling has been implicated in the mitigation of oxidative stress, suppression of cell death, and modulation of inflammatory responses.^16^

In addition to heat stress, mechanical force has also been reported to induce HSP expression or HSF activation in vascular smooth muscle cells, myocardium, and zebrafish embryos.^17^, ^18^, ^19^ In the lung, HSF1 activation ameliorates endotoxin-induced acute lung injury by attenuating inflammatory responses.^20^, ^21^ Despite these insights, the contribution of HSF1-mediated HSR to stretch-induced AEC injury in VILI remains unclear. We hypothesized that cyclic mechanical stretch activates HSF1-HSP signaling in AECs, thereby limiting oxidative damage and preserving cellular viability. To test this hypothesis, we employed an in vitro biaxial stretch model and combined transcriptomic, genetic, and pharmacological approaches to dissect the role of HSF1 in mechanically stretched AECs.

## Methods

### Cell culture

The A549 cell line was obtained from Shanghai Cell Bank (Chinese Academy of Sciences). A549 cells were cultured in RPMI-1640 medium (KGM31800; KeyGEN Biotech, China) supplemented with 10% fetal bovine serum (FBS; FSP500; ExCell, China), at 37°C and 5% CO_2_ incubator. The medium was changed every 2 or 3 days. Cells were passaged at 80% confluency and used between third and sixth passages in the following experiments.

### Mechanical stretch

Cells were seeded onto collagen I-coated elastic cell-stretching membranes at a density of 5×10^5^ cells per well and cultured for 24-48 h. The membranes were then mounted into the Biaxial Tank cell stretch chamber. Cells were divided into control and stretched groups. The stretched group was subjected to cyclic biaxial mechanical stretch using a Biaxial Tank cell stretch system. In this system, the elastic membrane carrying adherent cells is fixed within the stretch chamber and mechanically coupled to a motor-driven module. Programmed motor displacement deforms the membrane, thereby generating cyclic biaxial tensile strain that is transmitted to the adherent cells. The stretch parameters were set as follows: 0.5 Hz frequency, sine wave, and 10%, 20%, or 30% elongation for 6 h. These conditions were used to simulate pathological mechanical stress related to airway overdistension. The control group was cultured on the same elastic membranes and maintained in the same 37°C, 5% CO_2_ incubator without stretch stimulation.

### RNA extraction and quantitative real-time PCR (RT-qPCR)

FastPure Cell/Tissue Total RNA Isolation Kit V2 (RC112–1; Vazyme, China) was used to extract total RNA from control and stretched A549 cells. The quality and concentration of the extracted RNA were detected by Nucleic acid quantifier (NanoDrop One; Thermo, USA). cDNA was synthesized from all RNA samples using HiScript IIQ RT SuperMix for qPCR kit (R223–01; Vazyme, China) according to the manufacturer’s protocol. Expression of target gene mRNA in A549 cells was detected.

by RT-qPCR. RT-qPCR detection was performed using the Applied Biosystems 7500 Real-Time PCR System with ChamQ/SYBR qPCR Master Mix (Q331–02; Vazyme, China. RT-qPCR conditions were as follows: 40 cycles of predenaturation at 95°C for 30 s, denaturation at 95°C for 10 s, annealing at 60°C for 20 s, and extension at 72°C for 20 s. β-actin was used as the endogenous control gene, and expression levels of target genes were calculated by 2^-△△CT^ method after the reaction. Primer sequences for RT-qPCR are provided in the additional files (Table S4).

### Western blot

Cell were lysed with radioimmunoprecipitation assay lysates (P0013B；Beyotime) containing protease and phosphatase inhibitors, and the supernatant was collected after ultrasound and centrifugation. Protein concentrations were measured using a bicinchoninic acid protein quantification kit (P0012；Beyotime), and the equal amount of protein was mixed with 5× sodium dodecyl sulfate (SDS) loading buffer, heated at 95°C for 5 min, separated by 10% SDS–polyacrylamide gel electrophoresis. Proteins were transferred to polyvinylidene difluoride membrane (Millipore), blocked with 5% non-fat milk in TBST for 2 h at room temperature, and incubated overnight at 4°C with the primary antibodies: anti-Phospho-YAP (Ser127) (13008S; CST), anti-phospho-HSF1 (Ser326) (bsm-52166R; Bioss), anti-HSP70 (SOBO745; STARTER), anti-HSPA6 (68257-1-Ig; Proteintech), anti-YAP1 (13584-1-AP; Proteintech), anti-HSF1 (4356T; CST), anti-IL6 (WL02841; Wanleibiology), and anti-β-actin (66009-1-Ig; Proteintech) antibody. After being washed with TBST three times, membranes were incubated with horseradish peroxidase-labeled secondary antibody: anti-rabbit (SA00001-2; Proteintech) or anti-mouse IgG (SA00001-1; Proteintech), for 1 h at room temperature. Immunoreactive bands were visualized using ECL reagent (P10060; Beyotime) and captured with a chemiluminescence imaging system (ChemiDoc™ XRS+, Bio-Rad). Band intensities were quantified with ImageJ and normalized to β-actin. All experiments were repeated at least three times in each group.

### Immunofluorescence

Cells were rinsed twice with ice-cold PBS and fixed with 4% paraformaldehyde (PFA; VIH100; VICMED, China) at room temperature for 20 min; The PFA was washed away twice with PBS. The subsequent permeabilization was performed by incubating cells with 0.1%Triton X-100 (ST797; Beyotime, China) in PBS for 10 min. Cells were blocked with 1% BSA (VIC018; VICMED, China) in PBS at room temperature for 1 h and incubated overnight at 4°C with anti-YAP primary antibody (1:500, 13584-1-AP; Proteintech, China). After washing with PBS, cells were stained with Alexa Fluor-conjugated secondary antibody for 1 h at room temperature and counterstained with 4′,6-diamidino-2-phenylindole (DAPI, 1:1000) (40728ES03; Biosharp, China) for 10 min in the dark. The stained cells attached to the silicone membranes were cut off the plates and mounted inverted on slides using the anti-fluorescence attenuation tablet (P0126; Beyotime, China). Immunofluorescence images were acquired with fluorescence microscopy (Olympus; Tokyo, Japan).

### RNA-sequence and data analysis

We harvested 5×10^5^ A549 cells from unstretched and 6 h stretched groups to perform mRNA-Seq. Every group contained three biological replicates. Total RNA was extracted using a Trizol reagent kit (R401–01; VICMED, China) according to the manufacturer’s protocol. The isolated RNA samples were sent to Shanghai Zhengneng Biological Company for library construction and subsequent sequencing analysis. High-throughput sequencing was performed using the Illumina Novaseq 6000 platform to generate raw sequencing data. To ensure consistency between all samples, data normalization and bioinformatics analysis were performed using R software, version 4.3.2. Different expressed genes (DEGs) were identified by setting a statistical threshold (|log_2_ (FC)| > 1 and *P* adj value < 0.05) so as to determine the genes with significant differences between the two groups.

Gene set enrichment analysis (GSEA). Pre-ranked GSEA was performed using the GSEA software (v4.3.2, Broad Institute) with the following curated gene sets: (i) HSF1 heat-shock targets and HSF1 oxidative-stress-associated targets, derived from published PRO-seq and ChIP-seq data identifying HSF1-bound genes under heat shock (HS) and menadione-induced oxidative stress (MD) conditions^22^; (ii) the HSF1/HSF2 cancer signature, derived from shared chromatin occupancy and common transcriptional targets of HSF1 and HSF2 identified across diverse cancer cell lines^23^; and (iii) YAP/TEAD targets, compiled from the MSigDB C3 TFT (Transcription Factor Targets) collection (v7.2), specifically the YAP1_Q1, TEAD1_Q1, and TEAD4_Q1 gene sets.^24^ For HSF1 stress-responsive gene sets, mouse gene symbols from the original supplementary tables were converted to human orthologs using the HUGO Gene Nomenclature Committee comparative genomics resource; non-orthologous genes were excluded, yielding 73 genes for the HSF1 heat-shock set and 32 genes for the HSF1 oxidative-stress set. For the HSF1/HSF2 cancer signature, genes were extracted from published heat maps and figure descriptions,^23^ combined with canonical HSF1 cancer-program targets, and deduplicated to yield 28 genes. For YAP/TEAD targets, the three MSigDB subsets were merged and deduplicated to yield 35 genes. Genes were ranked by the signal-to-noise metric computed from the RNA-seq differential expression analysis (stretched vs. static). Enrichment was assessed using 1000 gene-set permutations, and significance was defined by normalized enrichment score (NES) and false discovery rate (FDR) < 0.05. Overlap and hypergeometric enrichment analyses between stretch-upregulated genes and each gene set were performed using the R package “GeneOverlap” (v1.36.0).

### Reactive oxygen species (ROS) free radical detection

After treatment, cells were washed twice with PBS and loaded with 10 μM 2′, 7′-Dichlorodihydrofluorescein diacetate (DCFH-DA) (KGA7308; KeyGEN Biotech, China) diluted 1:1000 in serum-free RPMI-1640 medium (KGM31800; The KeyGEN Biotech, China). After 30 min incubation at 37°C in the dark, cells were rinsed three times with PBS and maintained in 1 mL serum-free medium. Finally, fluorescent images were captured by fluorescence microscopy (Olympus; Tokyo, Japan), and image processing was performed by Image-J. The experiments were repeated at least three times in each group.

### Propidium iodide (PI) staining

After treatment, 2 μL 10 ug/mL PI (HY-D0815; MCE, China) was added to each well and incubated for 5 min at room temperature in the dark. Cell fluorescence images were observed using a fluorescence microscope. Image processing was performed by Image-J. The experiments were repeated at least three times in each group.

### HSF1 inhibitor

A549 cells were seeded onto collagen-I-coated culture dishes and cultured for 24 h. Then cells were treated for 1 h with 5 μM DTHIB (HY-138280; MCE, China)^25^ or the same volume of DMSO (12.VIC148; VICMED, China) before initiation of mechanical stretch.

### Generation and validation of HSF1-knockout A549 cells by CRISPR/Cas9

HSF1-knockout A549 cells were generated using the QuickEdit™ CRISPR/Cas9 knockout kit (Q051208; Base Therapeutics, Shanghai, China) according to the manufacturer’s instructions. This system delivers modified Cas9 mRNA together with a chemically synthesized single-guide RNA (sgRNA) into cultured adherent mammalian cells. After delivery, Cas9 mRNA is translated into Cas9 nuclease, which is guided by the sgRNA to the target HSF1 genomic sequence and induces DNA double-strand breaks. These breaks are repaired mainly through non-homologous end joining, resulting in insertion or deletion mutations and gene disruption. The sgRNA targeting human HSF1 was designed against an NGG protospacer-adjacent motif-compatible sequence, and the sgRNA sequence is provided in Supplementary Table S3. A non-targeting sgRNA was used as the negative control. Briefly, A549 cells were seeded in 12-well plates and transfected when they reached 60%–80% confluence. Before transfection, the medium was replaced with fresh serum-free medium. The Cas9 mRNA/sgRNA transfection complex was prepared according to the manufacturer’s protocol and directly added to the cells, followed by gentle shaking to ensure even distribution. After 4–6 h of transfection, the medium was replaced with fresh complete medium containing serum. At 48–72 h after transfection, cells were collected and subjected to limiting dilution to isolate single-cell-derived clones. Individual clones were expanded and screened for HSF1 knockout efficiency. HSF1 depletion was evaluated by Western blotting.

### Statistical analysis

All statistical analyses were performed using GraphPad Prism 9 (GraphPad Software, San Diego, CA, USA). Data are presented as the mean ± standard deviation. The unpaired *t* test was used to compare the data of two groups, and one-way ANOVA was used to compare the differences between the sample means of multiple groups. *P*< 0.05 was considered statistically significant.

## Results

### Construction of pathological mechanical stretching model

**Fig. 1:**
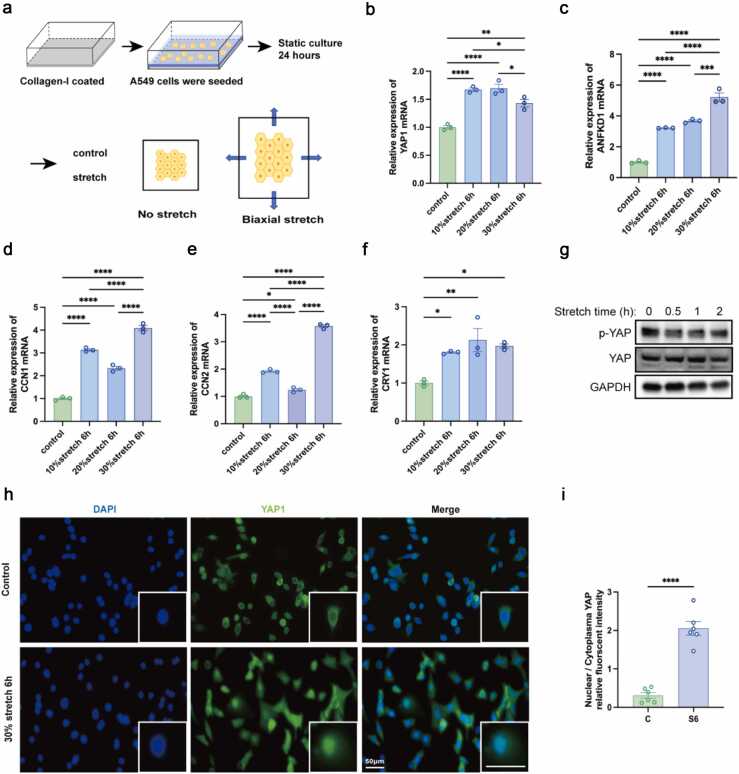
Biaxial cyclic stretch induces amplitude-dependent activation of the YAP signaling pathway in A549 cells. (a) schematic illustration of the biaxial mechanical stretch regimen. (b-f) Cells were subjected to cyclic stretch (0.5 Hz, sine waveform) at 10%, 20%, or 30% elongation for 6 h. RT-qPCR demonstrates the mRNA levels of *YAP* and its downstream targets *ANKRD1, CCN1, CCN2*, and *CRY1* in the control group and the stretching groups. (g) Western blot shows the expression of phosphorylated YAP protein in static controls and cells exposed to 30% stretch for 0.5, 1, and 2 h. (h) Confocal micrographs illustrated YAP subcellular distribution in static control and 30% stretch-treated (6 h) A549 cells. DAPI (blue) and YAP (green) channels are shown separately, along with merged images (right panels). Scale bar: 50 μm; *n* = 3. (i) Quantitative ImageJ analysis of the nuclear-to-cytoplasmic fluorescence intensity ratio of YAP (*n* = 3). C, static control; S2, 30% stretch for 2 h; S6, 30% stretch for 6 h. (Compared with the control group, * *P* < 0.05, ** *P* < 0.01, *** *P*< 0.001, **** *P* < 0.0001, ns: no statistical difference).

We evaluated the effects of different stretch amplitudes on YAP signaling activation in A549 cells. A schematic diagram of the biaxial stretch configuration is shown in Figure 1a. Compared with static controls, mechanical stretch significantly increased the mRNA expression of YAP and its downstream target genes, including ANKRD1, CCN1, CCN2, and CRY1, in A549 cells (*P* < 0.05). Among the tested stretch amplitudes, 30% elongation induced a more prominent YAP-associated transcriptional response than 10% or 20% elongation (Figure 1b-f). Consistently, Western blot analysis showed that cyclic stretch at 30% elongation significantly reduced phosphorylated YAP levels at 0.5, 1, and 2 h (Fig. 1G), supporting stretch-induced activation of YAP signaling. Immunofluorescence analysis further demonstrated that, under static conditions, YAP was predominantly sequestered in the cytoplasm, whereas 6 h of mechanical stretch induced pronounced nuclear accumulation of YAP (Figure 1h). Quantitative assessment of subcellular fluorescence intensity confirmed an increased nuclear-to-cytoplasmic YAP ratio under stretch (*P* < 0.05, Figure 1i). Together, these results indicate that 30% cyclic stretch effectively activates mechanotransduction-related YAP signaling in A549 cells and was therefore selected for subsequent experiments.

### Mechanical stretch rapidly increases intracellular ROS levels but does not induce significant cell death within 6 h

**Fig. 2:**
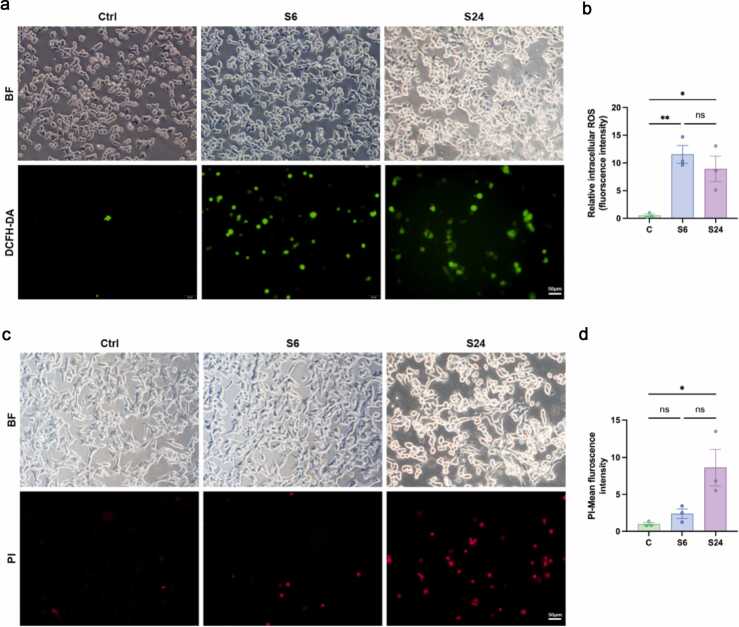
Cyclic mechanical stretch rapidly elevates intracellular ROS without significant early cell death in A549 cells. (a) Representative fluorescence micrographs of DCFH-DA staining showed ROS production in static controls and after 30% cyclic stretch for 6 or 24 h. (b) Quantitative analysis of DCFH-DA fluorescence intensity (mean ± SD; *n* = 3; *P< 0.05, **P < 0.01 vs. control). (c) Propidium iodide (PI) staining indicated membrane-permeable (dead) cells under identical experimental conditions. (d) Quantification of PI-positive nuclei (mean ± SD; *n* = 3; *P < .05, ns: no statistical difference：stretch vs. control).

Exposure to 30% cyclic stretch elicited a marked rise in ROS generation, as detected by DCFH-DA, after both 6 and 24 h compared with static controls (*P*< 0.05, Figure 2a, b). In parallel, PI staining revealed no significant increase in cell death at 6 h (Figure 2c, d). Only after prolonged stretch (24 h) was a pronounced increase in PI-positive nuclei observed (*P* < 0.05, Figure 2c, d), indicating that short-term mechanical stress elevates oxidative burden without immediate cytotoxicity.

### Transcriptomic analysis of stretch-induced gene expression changes in A549 cells

**Fig. 3:**
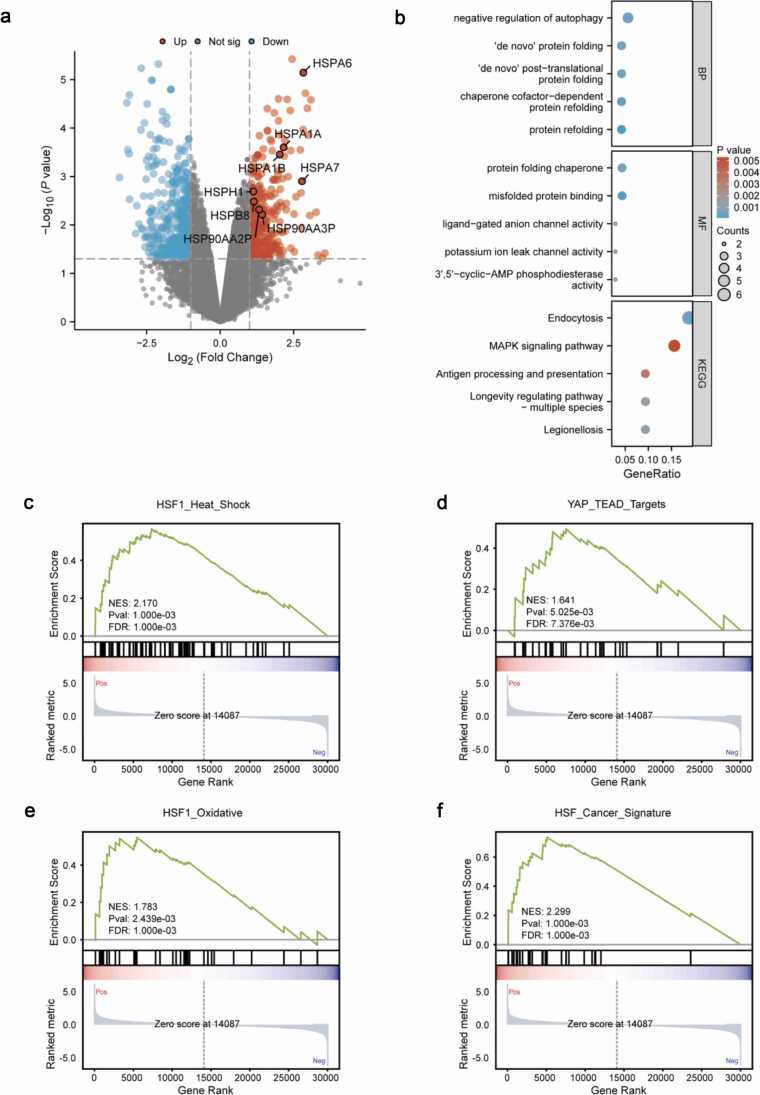
Mechanical stretch promotes the upregulation of heat shock protein expression. (a) Volcano plot of differentially expressed genes (DEGs) in stretched versus static A549 cells (|log₂FC= ≥ 1, FDR < 0.05). Down-regulated genes are depicted in blue, up-regulated genes in red, and non-significant genes in gray. Key up-regulated heat-shock protein genes are highlighted. (b) GO and KEGG pathway enrichment analyses of DEGs reveal significant over-representation of “chaperone-mediated protein refolding” and related proteostasis pathways. (c-f) Pre-ranked GSEA was performed using the genome-wide ranked RNA-seq gene list from cyclic stretch-treated A549 cells versus static controls. Enrichment plots are shown for HSF1 heat-shock targets^22^ (c), YAP/TEAD targets^24^ (d), HSF1 oxidative-stress-associated targets^22^ (e), and the HSF1/HSF2 cancer signature^23^ (f). Positive NES values indicate enrichment toward genes upregulated by cyclic stretch.

To elucidate the transcriptional landscape evoked by pathological mechanical stretch, RNA-seq was performed using three biological replicates from A549 cells exposed to 30% cyclic stretch for 6 h and three biological replicates from unstretched control cells. Genes with a FDR < 0.05 and |log₂FC= ≥ 1 were identified as DEGs. In total, 754 DEGs were identified between stretched (S) and unstretched (N) groups (Figure 3a). The complete list of DEGs is provided in Table S1. Among the significantly upregulated DEGs, several heat-shock protein (HSP)-related genes were identified, including *HSPA1A, HSPA1B, HSPA6, HSPA7, HSPB8, HSPH1, and HSP90AA2P* (Figure 3a and Table S2).

Gene Ontology enrichment analysis was performed to characterize the biological functions associated with stretch-induced DEGs. Biological process analysis revealed significant enrichment of terms related to negative regulation of autophagy and chaperone cofactor-dependent protein refolding. Molecular function analysis indicated enrichment of genes primarily involved in protein binding, ATP binding, and related molecular activities. Kyoto Encyclopedia of Genes and Genomes pathway analysis showed that the DEGs were mainly enriched in endocytosis, the mitogen-activated protein kinase signaling pathway, and the longevity regulating pathway across multiple species (Figure 3b).

To further assess the contribution of HSF and YAP/TEAD-associated transcriptional programs to the stretch-induced transcriptomic response, pre-ranked GSEA was performed using the genome-wide ranked RNA-seq gene list from stretched versus unstretched A549 cells. Four curated gene sets were analyzed, including HSF1 heat-shock targets, HSF1 oxidative-stress-associated targets (derived from PRO-seq and ChIP-seq data under heat shock and menadione-induced oxidative stress^22^), the HSF1/HSF2 cancer signature (derived from shared chromatin occupancy and transcriptional targets of HSF1 and HSF2 in cancer cells^23^), and YAP/TEAD targets (compiled from MSigDB C3 TFT transcription factor target gene sets^24^). HSF1 heat-shock targets were significantly enriched in stretch-treated cells (NES = 2.170, FDR = 1.000 × 10^−3^; Figure 3c). YAP/TEAD targets were also significantly enriched after stretch, although with a lower NES value (NES = 1.641, FDR = 7.376 × 10^−3^; Figure 3d). HSF1 oxidative-stress-associated targets and the HSF1/HSF2 cancer signature were likewise enriched, with NES values of 1.783 and 2.299, respectively (Figure 3e and f). The detailed GSEA results, including NES, nominal *P* values, FDR values, and leading-edge genes, are provided in Table S5.

To further compare the representation of HSF- and YAP/TEAD-related targets among stretch-induced genes, overlap and hypergeometric enrichment analyses were performed. HSF1 heat-shock targets were significantly over-represented among stretch-upregulated genes, whereas YAP/TEAD targets showed limited overlap and were not significantly enriched in this subset. Detailed overlap genes and hypergeometric enrichment statistics are provided in Tables S6 and S7. Together, these findings indicate that cyclic stretch induces both HSF- and YAP/TEAD-related transcriptional responses, with a more prominent enrichment of HSF-associated chaperone gene signatures among the most strongly stretch-induced genes.

### Mechanical stretch induces HSF1 activation and heat-shock protein expression in epithelial cells

**Fig. 4:**
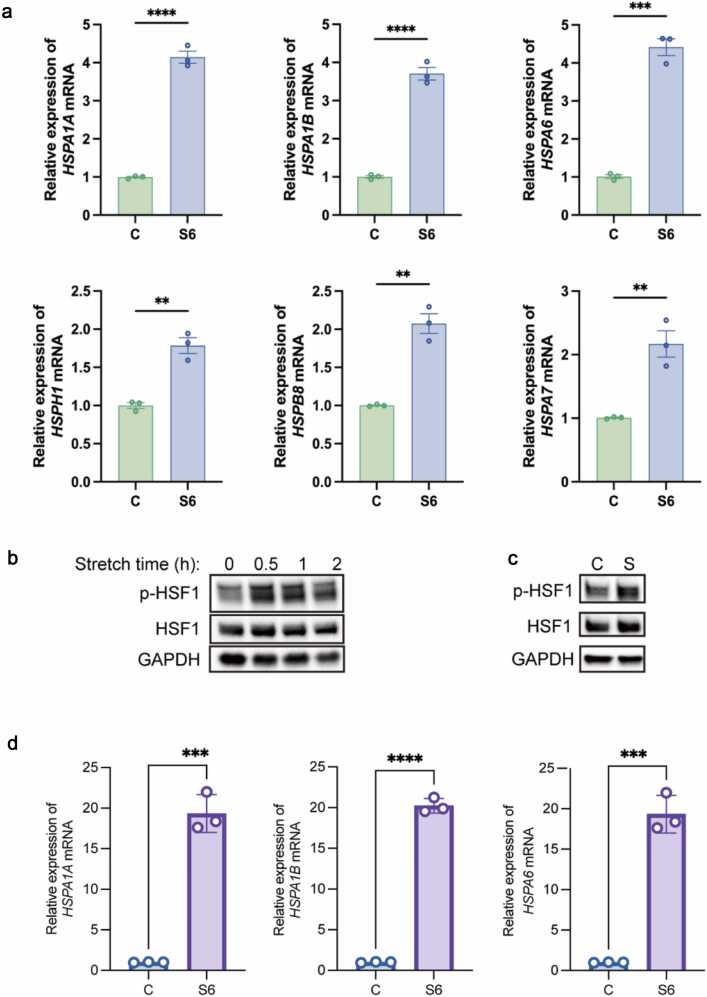
Mechanical stretch induces HSF1 activation and heat-shock protein expression in epithelial cells. (a) RT-qPCR validation of mRNA-seq data confirms significant up-regulation of *HSPA1A*, *HSPA1B*, *HSPA6*, *HSPA7*, *HSPB8*, and *HSPH1* after 6 h of 30% cyclic stretch (mean ± SD, *n* = 3; *P< 0.05, **P < 0.01, ***P< 0.001 vs. control). (b) Western blot analysis of phosphorylated HSF1 and total HSF1 protein levels in A549 cells after 30% cyclic stretch for the indicated time points. (c) Western blot analysis of phosphorylated HSF1 and total HSF1 protein levels in BEAS-2B cells after cyclic stretch for 1 h. (d) RT-qPCR analysis of HSPA1A, HSPA1B, and HSPA6 mRNA expression in BEAS-2B cells exposed to 30% cyclic stretch for 6 h.

To verify the results of our sequencing data, we quantified the mRNA expression levels of related HSPs genes, including *HSPA1A, HSPA1B, HSPA6, HSPA7, HSPB8, and HSPH1*, using RT-qPCR. Consistent with transcriptome sequencing data, 6 h of 30% cyclic stretch significantly increased mRNA levels of the above-mentioned genes contrast to the control group (*P* < 0.05, Figure 4a).

Since HSF1 is a key transcription factor involved in the induction of a subset of stress-inducible HSPs during the HSR, we hypothesized that HSF1 may contribute to the stretch-induced HSP expression program in AECs. Western blot analysis showed that cyclic stretch at 30% elongation increased HSF1 phosphorylation in A549 cells, while total HSF1 protein levels remained relatively unchanged during the examined time course (Figures 4b and S1). To further assess the generalizability of this response, we examined HSF1 activation in BEAS-2B bronchial epithelial cells. Similarly, cyclic stretch increased HSF1 phosphorylation in BEAS-2B cells (Figure 4c). Moreover, 30% cyclic stretch for 6 h significantly upregulated the mRNA expression of HSPA1A, HSPA1B, and HSPA6 in BEAS-2B cells (*P* < 0.05, Figure 4d). Together, these results indicate that cyclic stretch activates HSF1 signaling and induces HSP expression in airway epithelial cell models.

### Pharmacological inhibition of HSF1 attenuates stretch-induced HSP expression

**Fig. 5:**
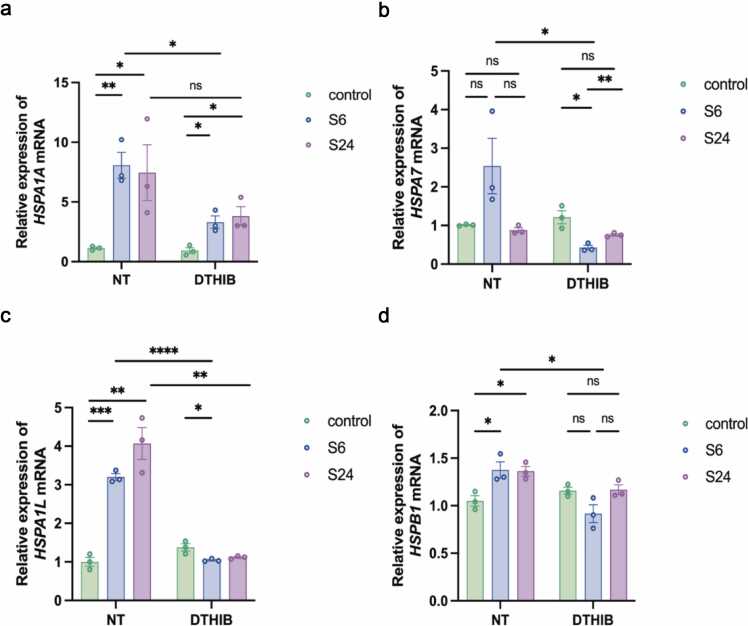
Pharmacological inhibition of HSF1 attenuates cyclic stretch-induced HSP expression. (a-d) RT-qPCR validation of stretch- and HSF1-dependent HSP transcription. Cells were pre-treated with 5 µM DTHIB or the same volume DMSO vehicle for 1 h prior to 30% cyclic stretch. DTHIB significantly blunted stretch-induced up-regulation of *HSPA1A*, *HSPA1L*, *HSPA7*, and *HSPB1* mRNA, with maximal inhibition observed at 6 h (mean ± SD, *n* = 3；C, static control; S6, 30% stretch for 6 h; S24, 30% stretch for 24 h. NT, no treated, only added to the same volume DMSO) (*P < 0.05, **P < 0.01, ***P< 0.001, ****P< 0.0001，ns, not significance vs. stretch-only).

To substantiate the functional contribution of HSF1, A549 cells were pretreated for 1 h with the selective HSF1 inhibitor DTHIB (5 μM), which promotes nuclear HSF1 degradation, before mechanical stimulation. RT-qPCR analysis showed that cyclic stretch increased *HSPA1A*, *HSPA1L*, *HSPA7*, and *HSPB1* mRNA levels, with most genes showing peak induction at 6 h. DTHIB markedly attenuated stretch-induced expression of *HSPA1A*, *HSPA1L*, *HSPA7*, and *HSPB1*, with the inhibitory effect most pronounced at 6 h (*P* < 0.05 vs. stretch alone; Figure 5a-d). DTHIB alone did not markedly alter basal HSP expression, supporting the contribution of HSF1-dependent transcriptional activation during mechanical stress.

### Oxidative stress-dependent HSF1 activation mediates stretch-induced HSP expression and cytoprotection

**Fig. 6:**
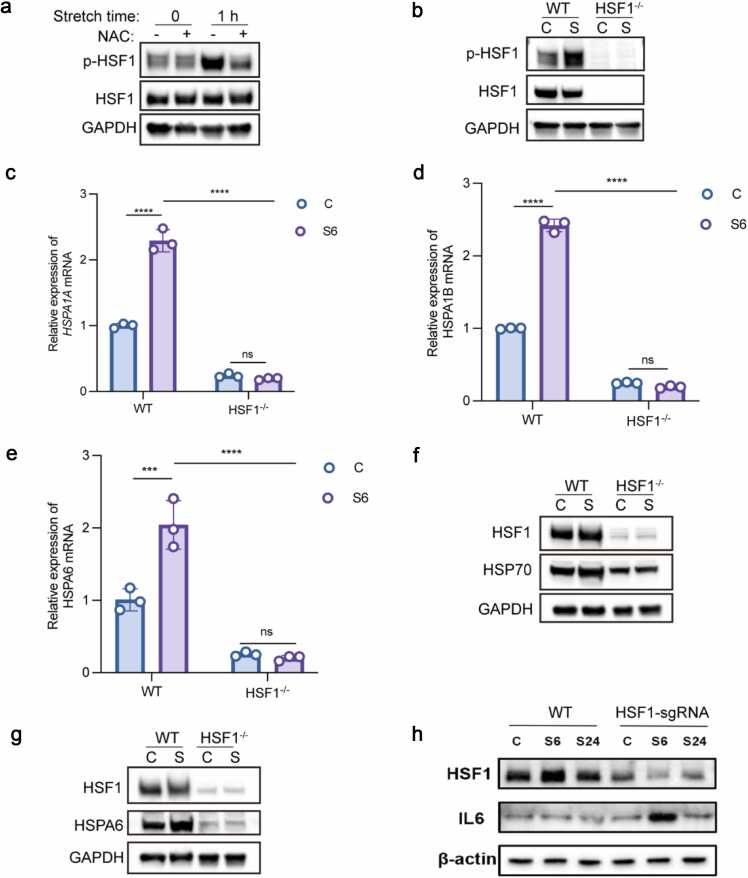
*Oxidative stress-dependent HSF1 activation mediates stretch-induced HSP expression and limits IL-6 production*. (a) NAC (5 mM) pretreatment for 1 h attenuated cyclic stretch-induced HSF1 phosphorylation in A549 cells, as assessed by Western blot. (b) Western blot validation of HSF1 depletion in CRISPR/Cas9-generated HSF1^-/-^ A549 cells. (c-e) RT-qPCR analysis of (c) *HSPA1A*, (d) *HSPA1B* and (e) *HSPA6* mRNA levels after 6 h of 30% cyclic stretch. (mean ± SD, *n* = 3; *P < 0.05, **P < 0.01, ***P< 0.001, vs. WT cells). (f-g) Western blot analysis showing that stretch-induced increases in HSP70 and HSPA6 protein levels were attenuated in HSF1^-/-^ A549 cells. (h) Western blots shows the expression of HSF1 protein and IL-6 protein in wild-type (WT) and HSF1-sgRNA cells under static control conditions or after 30% cyclic stretch for 6 and 24 h.

Because cyclic stretch increased ROS production in A549 cells, we next examined whether oxidative stress contributed to stretch-induced HSF1 activation. Pretreatment with the antioxidant N-acetyl-L-cysteine (NAC) markedly attenuated stretch-induced HSF1 phosphorylation, whereas total HSF1 protein levels remained largely unchanged (Figure 6a). These findings suggest that oxidative stress acts upstream of HSF1 activation in response to cyclic stretch.

We next asked whether previously reported HSF1 regulatory mechanisms might also contribute to this response. Previous genome-wide regulatory studies identified EP300 and the proteasome system as regulators of the activatable HSF1 pool and JMJD6 as a positive regulator of HSF1 activity.^26^, ^27^ We therefore pretreated A549 cells with the EP300 inhibitor A-485 or the JMJD6 inhibitor SKLB325 before cyclic stretch. A-485 had no significant effect on stretch-induced upregulation of HSPA1A, HSPA1B, or HSPA6. In contrast, SKLB325 modestly but significantly reduced stretch-induced HSPA1A and HSPA1B expression, whereas its effect on HSPA6 was not statistically significant (Figure S1). However, HSP gene expression remained substantially elevated after SKLB325 treatment compared with static controls. These results suggest that JMJD6 may partially contribute to stretch-induced HSP gene induction, but JMJD6-dependent regulation is unlikely to be the dominant mechanism driving HSF1 activation in this model. Together with the NAC data, these findings support a model in which stretch-induced oxidative stress contributes to HSF1 activation.

To genetically validate the role of HSF1 in stretch-induced HSP expression, we generated single-cell-derived HSF1-knockout A549 clones using CRISPR/Cas9. Western blot analysis verified efficient depletion of HSF1 protein in the selected HSF1-KO clone (Figure 6b). Under 30% cyclic stretch, WT A549 cells exhibited robust HSF1 phosphorylation and marked induction of HSP gene expression. In contrast, HSF1-KO cells showed substantially attenuated induction of HSPA1A, HSPA1B, and HSPA6 transcripts after cyclic stretch compared with WT cells (*P* < 0.05 vs. WT-stretch; Figure 6c-e). Consistently, the protein levels of HSP70, encoded by HSPA1A and HSPA1B, and HSPA6 were markedly increased after stretch in WT cells but were reduced in HSF1-KO cells (Figure 6f and g). These results confirm that stretch-induced HSP expression is largely dependent on HSF1.

We further assessed whether HSF1 disruption affected inflammatory and injury-related responses under mechanical stress. Because these functional injury assays were performed using pooled HSF1-sgRNA cells generated in the original CRISPR/Cas9 experiments, we describe these results separately from the single-cell-derived HSF1-KO clone validation. In WT A549 cells, 6 or 24 h of 30% cyclic stretch did not significantly alter IL-6 protein levels relative to static controls. In contrast, pooled HSF1-sgRNA cells exhibited a pronounced increase in IL-6 protein after cyclic stretch compared with WT-stretch cells (*P* < 0.01; Figure 6h). These data suggest that partial disruption of HSF1 may enhance stretch-associated pro-inflammatory signaling in mechanically stressed AECs.

**Fig. 7:**
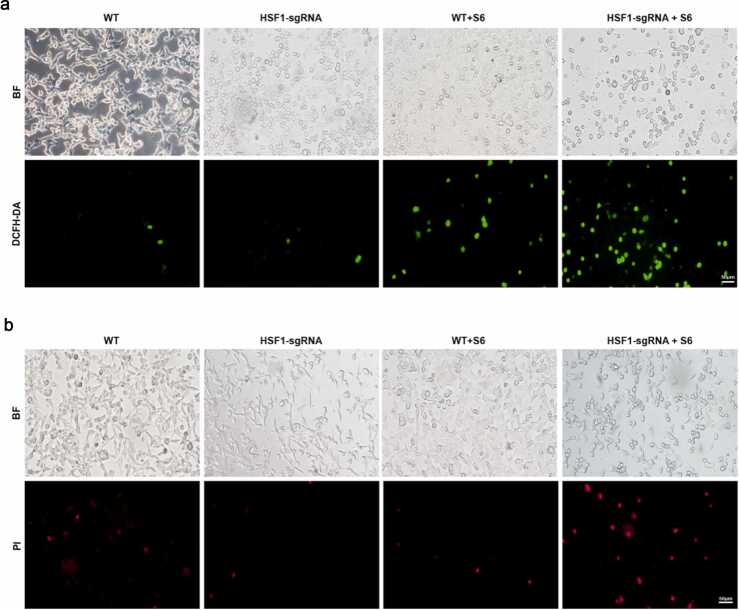
*HSF1 deletion exacerbates stretch-induced oxidative stress and cell death.* (a) Immunofluorescence images of DCFH-DA staining showed ROS production between wild-type (WT) cells and HSF1-sgRNA cells after 6 h of 30% cyclic stretch. (b) PI staining indicated membrane-permeable (dead) cells under identical experimental conditions. HSF1-sgRNA cells displayed significantly elevated ROS accumulation and PI-positive nuclei relative to WT under the mechanical stretching.

The functional consequences of HSF1 disruption were further assessed using DCFH-DA and PI staining. Baseline ROS levels and cell death were comparable between WT and pooled HSF1-sgRNA cells. However, after 6 h of cyclic stretch, pooled HSF1-sgRNA cells accumulated significantly higher levels of ROS and exhibited an increased number of PI-positive nuclei compared with WT cells (Figure 7a and b). Together, these findings indicate that stretch-induced oxidative stress contributes to HSF1 activation and that HSF1-dependent HSP induction may protect epithelial cells from excessive oxidative injury and cell death under pathological mechanical stretch.

## Discussion

Mechanical ventilation is indispensable for critically ill patients, yet it constitutes a double-edged sword: while providing life-sustaining respiratory support, it may also precipitate VILI.^28^ AECs, the fundamental structural and functional units of the alveolus, are continuously exposed to cyclic distension and recoil of the basement membrane during breathing and mechanical ventilation.^29^ Through specialized mechanosensors and intracellular transduction cascades, these physical forces are converted into biochemical signals that dictate cell fate and behavior.^30^ The Hippo pathway effector YAP functions as a prototypical mechanotransducer.^31^ Consistent with previous reports,^9^ our data demonstrate that cyclic stretch activates YAP signaling in a magnitude-dependent manner, as reflected by increased expression of YAP and its target genes, reduced YAP phosphorylation, and enhanced YAP nuclear localization. Among the tested stretch amplitudes, 30% biaxial stretch most robustly promoted YAP-associated transcriptional responses and was therefore selected for subsequent experiments to model VILI-associated pathological mechanical stress.

Oxidative stress arises when the generation of ROS exceeds antioxidant capacity.^13^ Excessive mechanical stretch or high-tidal-volume ventilation causes alveolar and endothelial overdistension, promotes ROS generation, and contributes to oxidative stress, barrier dysfunction, and cell death.^32^ Our data show that 30% cyclic stretch for 6 and 24 h markedly elevates intracellular ROS compared with static controls. These findings are consistent with previous studies showing that cyclic mechanical stretch promotes oxidative stress in AECs through NOX-dependent mechanisms^32^ and that biomechanical forces interact with oxidative stress pathways in pulmonary injury.^33^ ROS accumulation may also contribute to epithelial barrier dysfunction, as superoxide has been reported to mediate tight-junction complex dissociation in cyclically stretched lung tissue.^34^ Notably, while no overt cell death was observed after 6 h of stretch, PI-positive nuclei increased significantly at 24 h, suggesting that early oxidative stress precedes delayed epithelial injury and may trigger adaptive stress responses.

We therefore performed mRNA sequencing on A549 cells subjected to 6 h of stretch and on static controls. Both RNA-seq and RT-qPCR analyses revealed that mechanical stretch induces a rapid increase in HSP-related transcripts, including HSPA1A, HSPA1B, and HSPA6. This response is consistent with activation of the conserved HSR, whose hallmark is the induction of molecular chaperones that maintain proteostasis under stress conditions.^14^, ^35^ HSPs are molecular chaperones classified into several major families, including HSP110 family, HSP90 family, HSPA, HSP60/HSP10 chaperonin families, HSP40 family, and HSPB, according to standardized nomenclature guidelines.^15^ In this study, multiple HSP-related genes, particularly members of the HSPA and HSPB families, were upregulated after cyclic stretch, supporting the presence of an early adaptive chaperone response to pathological mechanical stimulation.

Among mammalian HSF paralogues, HSF1 is a key transcriptional regulator of stress-inducible HSP expression.^11^, ^12^ HSF1 also functions as a mechano-responsive transcription factor in several biological systems. Cyclic stretch of vascular smooth muscle cells induces HSP70 expression,^17^ ex vivo stretch of rat myocardium activates heat-shock factor through stretch-activated channels,^18^ and recent work in zebrafish embryos further supports the mechanosensitive nature of HSF1.^19^ In our study, cyclic stretch increased HSF1 phosphorylation, whereas total HSF1 protein levels remained relatively stable, indicating that stretch-induced HSF1 activation is likely mediated primarily through post-translational regulation rather than changes in total HSF1 abundance. Pharmacological inhibition or CRISPR/Cas9-mediated deletion of HSF1 markedly attenuated stretch-induced HSP expression, supporting an HSF1-dependent mechanism. Collectively, these findings demonstrate that mechanical stretch activates an acute HSF1–HSP cytoprotective program in epithelial cells.

HSF1–HSP signaling has been widely implicated in cellular adaptation to oxidative and inflammatory stress. HSP70 has been reported to reduce endotoxin-triggered ROS and TNF-α production in human phagocytes,^36^ and exosome-associated HSP70 suppresses ROS accumulation in cerebral ischemia/reperfusion injury.^37^ In lung injury models, HSF1-related responses have been associated with reduced inflammatory injury and improved tissue protection after endotoxin challenge.^20^, ^21^ Similar cytoprotective functions of HSF1 have also been reported in cardiomyocyte stress models.^38^ Consistent with these findings, we observed that HSF1 disruption enhanced ROS accumulation and PI-positive cell death after cyclic stretch. These data suggest that loss of HSF1 impairs the cellular stress response, weakens antioxidant defenses, and amplifies stretch-mediated injury.

Moreover, inflammatory amplification is an important component of VILI. HSP70 has been identified as a negative regulator of NLRP3 inflammasome activation and IL-1β secretion.^39^ In addition, HSP70 overexpression suppresses NF-κB signaling and attenuates LPS-induced production of pro-inflammatory cytokines.^40^ Consistent with a potential anti-inflammatory role of the HSR, we observed that IL-6 protein remained unchanged in stretched wild-type A549 cells, whereas pooled HSF1-sgRNA cells showed a marked increase in IL-6 production after cyclic stretch. These data suggest that HSF1 disruption may sensitize mechanically stressed epithelial cells to inflammatory signaling. Collectively, our findings position HSF1 as an endogenous stress-adaptive regulator that limits oxidative and inflammatory injury in epithelial cells subjected to pathological mechanical stretch.

Several limitations merit consideration. First, although key results were additionally validated in BEAS-2B cells, most mechanistic experiments were performed in A549 cells, a lung adenocarcinoma-derived epithelial cell line. Future investigations should incorporate primary AECs, lung organoids, and in vivo VILI models to confirm the physiological relevance of the HSF1–HSP axis. Second, although NAC partially suppressed stretch-induced HSF1 activation, the upstream activators linking mechanical stretch to HSF1 remain incompletely defined. Emerging evidence implicates Ca^2+^-related signaling in HSF1 activation,^41^ and intracellular Ca^2+^signaling is active in A549 cells.^42^ It is plausible that Ca^2+^-dependent or kinase-related pathways contribute to HSF1 activation under mechanical stretch, a hypothesis requiring future research. Third, we did not directly test whether YAP inhibition prevents stretch-induced HSF1 activation; therefore, possible YAP–HSF1 crosstalk cannot be excluded. Finally, although HSF1 activation may be cytoprotective in mechanically injured epithelial cells, sustained or excessive HSF1 activation has been implicated in tumor-associated stress adaptation and cancer progression.^43^, ^44^ Therefore, therapeutic strategies targeting HSF1 would require careful temporal and context-specific evaluation.

This study suggests that HSF1 may serve as an endogenous protective mechanism against mechanical stretch-induced AEC injury. Cyclic stretch induces oxidative stress and activates HSF1, leading to HSP induction that limits oxidative injury, inflammatory signaling, and cell death. These findings provide mechanistic insight into epithelial stress adaptation under pathological mechanical stretch and suggest that the HSF1–HSP axis may represent a potential target for lung-protective strategies in VILI. Further studies are needed to define the upstream mechanotransduction pathways leading to HSF1 activation and to validate this protective mechanism in primary epithelial cells and in vivo models.

## Conclusion

Our findings demonstrated that cyclic mechanical stretch rapidly activates the HSF1 signaling axis, driving an acute transcriptional program that up-regulates HSPs and confers cytoprotection against stretch-induced injury. Genetic or pharmacological inhibition of HSF1 abolished this response, resulting in excessive ROS accumulation, enhanced oxidative stress, and aggravated cell death. Collectively, these data confirmed HSF1 as an early endogenous defense mechanism that limits alveolar epithelial damage during pathological mechanical stress and highlight its potential as a therapeutic target for the prevention and mitigation of VILI.

## Ethics statement

This study exclusively involved in vitro experiments with cell lines and did not include human participants, animals, or patient-derived samples. Therefore, ethical approval, informed consent to participate, and consent to publish are not applicable.

## Funding and support

The work is supported by the Key Project of the Jiangsu Provincial Health Commission (K2024087) and Science Foundation of Kangda College of Nanjing Medical University (KD2023KYJJ035).

## CRediT authorship contribution statement

**Jinqiu Ding:** Writing – review & editing, Supervision, Project administration, Methodology, Investigation, Funding acquisition, Conceptualization. **Xinyi Tang:** Writing – review & editing, Writing – original draft, Formal analysis, Data curation, Conceptualization. **Haoyue Xue:** Writing – review & editing, Validation, Software, Resources, Methodology, Investigation, Data curation. **Zuxin Zhou:** Visualization, Validation, Software, Methodology. **Haoran Chen:** Visualization, Validation, Supervision, Software, Investigation, Formal analysis, Data curation. **Yao Yan:** Writing – review & editing, Supervision, Resources, Project administration, Investigation. **Yongpeng Xie:** Writing – review & editing, Supervision, Resources, Project administration, Funding acquisition.

### Declarations of interest

The authors declare that they have no known competing financial interests or personal relationships that could have appeared to influence the work reported in this paper.

## Data Availability

Data will be made available on request. Mendeley DataA549 biaxial stretch RNAseq

## Footnotes

Appendix A Supplementary data associated with this article can be found in the online version at doi:10.1016/j.cstres.2026.100196.
