# Corrigendum to “Heat shock transcription factor 1 acts as an endogenous protective mechanism in mechanically stretched alveolar epithelial cells” [Cell Stress and Chaperones 31 (2026) 100196]

**DOI:** 10.1016/j.cstres.2026.100206

**Published:** 2026-08-22

**Authors:** Jinqiu Ding, Xinyi Tang, Haoyue Xue, Zuxin Zhou, Haoran Chen, Yao Yan, Yongpeng Xie

**Affiliations:** 1 Department of Emergency Medicine, The Affiliated Lianyungang Hospital of Xuzhou Medical University, The Lianyungang Clinical College of Nanjing Medical University, The First Affiliated Hospital of Kangda College of Nanjing Medical University, The First People's Hospital of Lianyungang, Lianyungang, Jiangsu 222000, China; 2 Department of Intensive Care Medicine, Affiliated Hospital of Nantong University, Nantong, Jiangsu 226000, China

The authors regret that, during figure assembly, incorrect versions of the HSPA1B and HSPA6 plots were inadvertently inserted into Figure 4d. Consequently, the published panel does not accurately reflect the verified RT-qPCR data for these two genes. This correction is therefore necessary to ensure that the figure faithfully represents the underlying experimental data and that the scientific record is accurate. The corrected Figure 4d is provided as a separate file, and the figure legend remains unchanged. The corrected plots continue to show significant up-regulation of HSPA1B and HSPA6 after cyclic stretch; therefore, this correction does not affect the interpretation or conclusions of the article.

**Figure:**
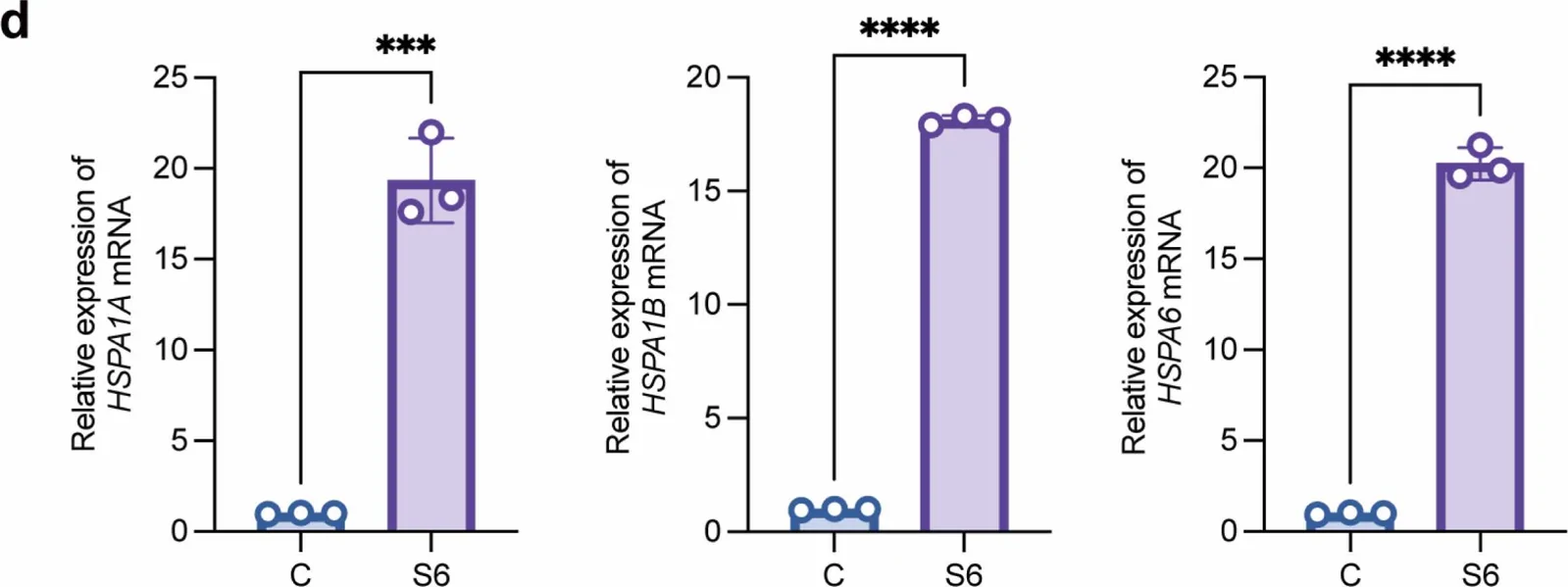

The authors would like to apologize for any inconvenience caused.

_______________________

