# Supplementary material for: Heat shock transcription factor 1 acts as an endogenous protective mechanism in mechanically stretched alveolar epithelial cells
Source: Cell Stress Chaperones. 2026 Jul 10;31(5):100196. doi: 10.1016/j.cstres.2026.100196 (PMC13427404; doi:10.1016/j.cstres.2026.100196)
Supplement: Supplementary file 1 — Supplementary material [file mmc1.docx]

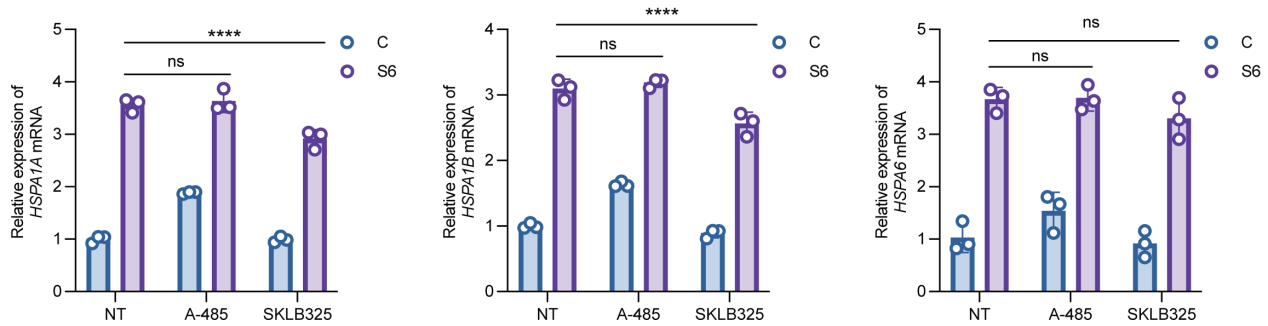


**Supplementary Figure S1. Pharmacological assessment of EP300 and JMJD6 in stretch-induced HSP gene expression.**
