# Supplementary figures and images for: Heat shock transcription factor 1 acts as an endogenous protective mechanism in mechanically stretched alveolar epithelial cells
Source: Cell Stress Chaperones. 2026 Jul 10;31(5):100196. doi: 10.1016/j.cstres.2026.100196 (PMC13427404; doi:10.1016/j.cstres.2026.100196)

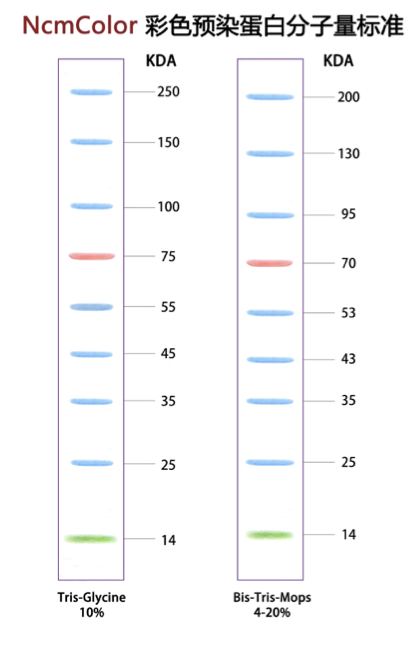


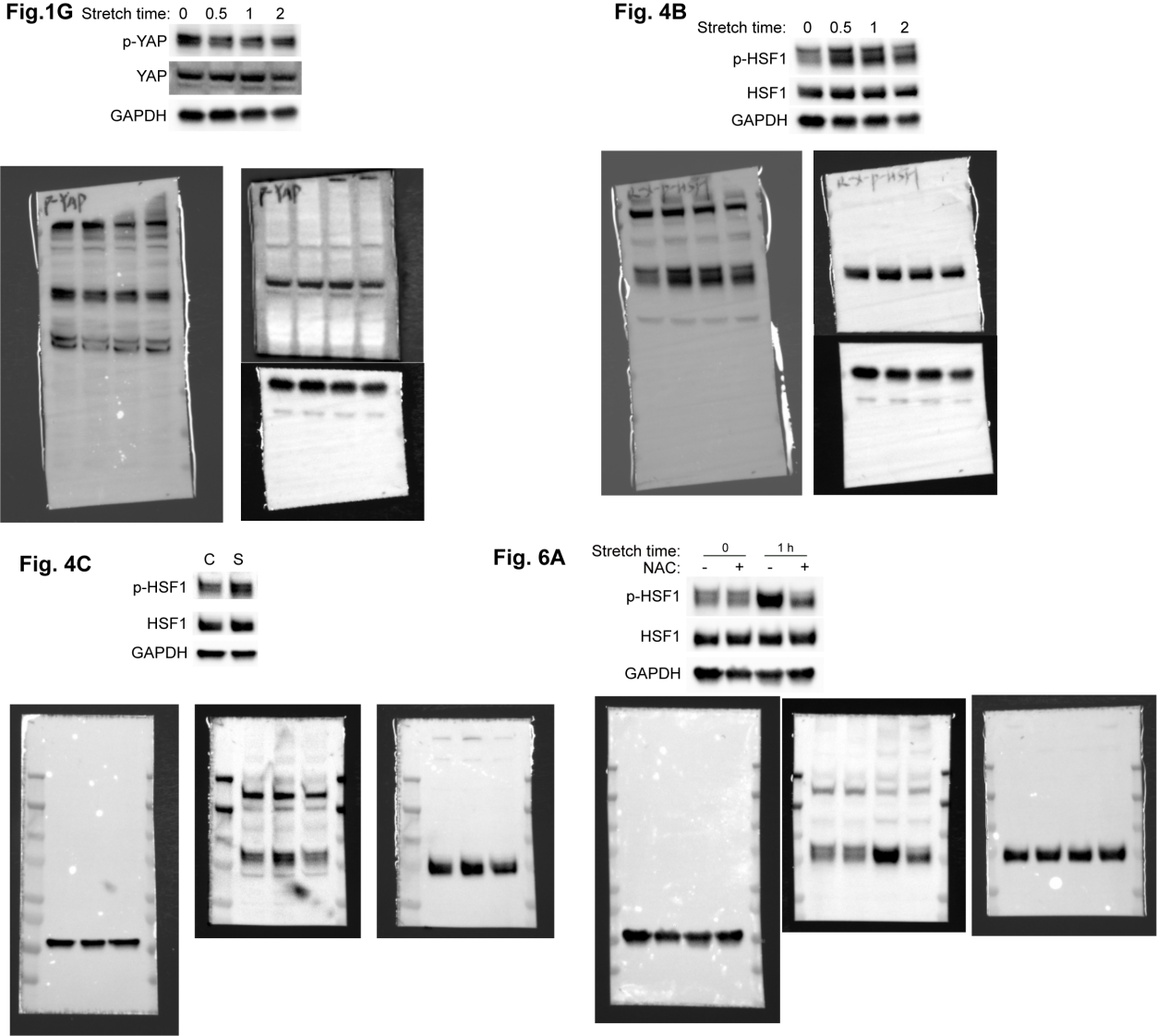


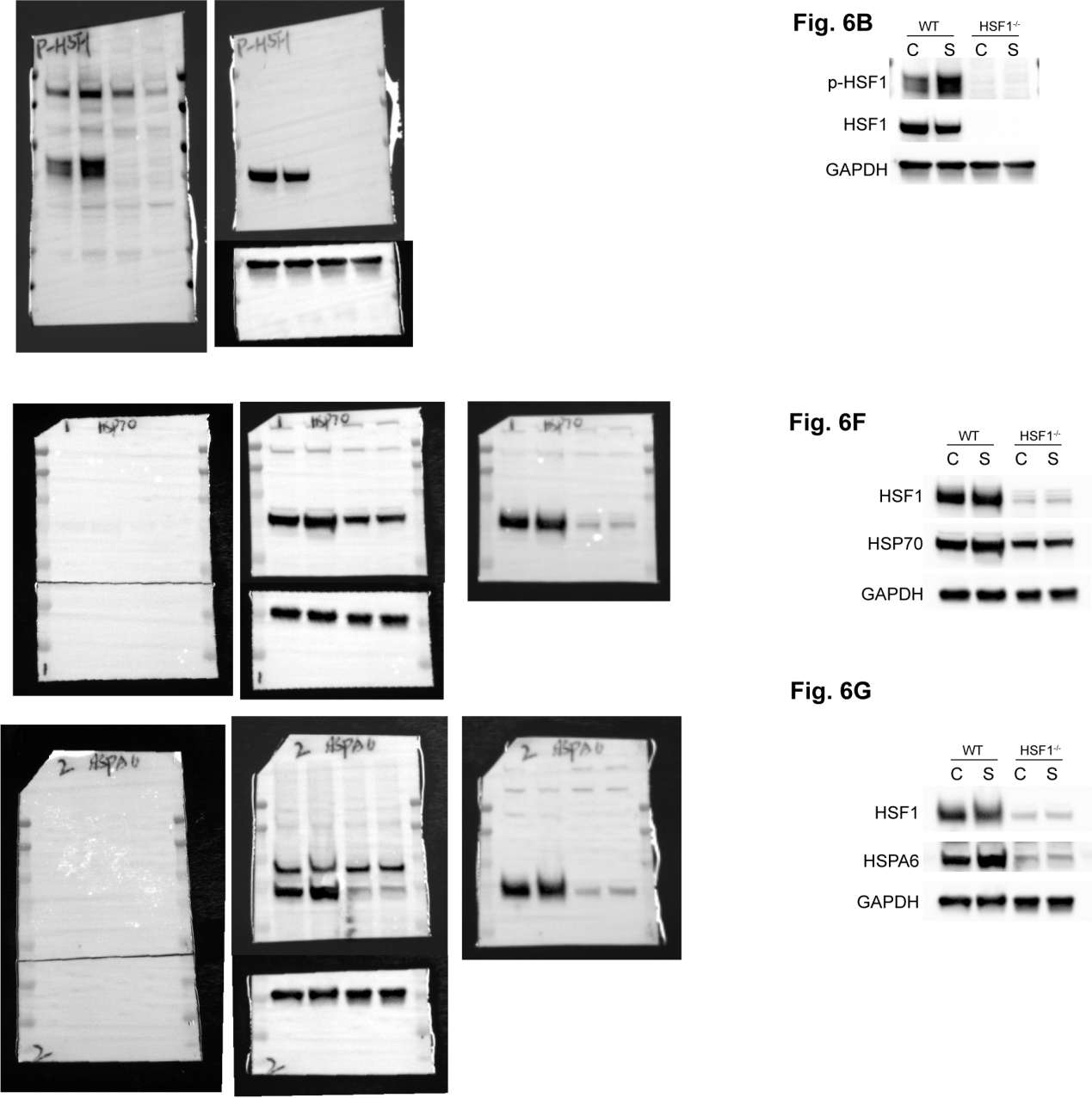


Fig.6H HSF1 and IL6 expression


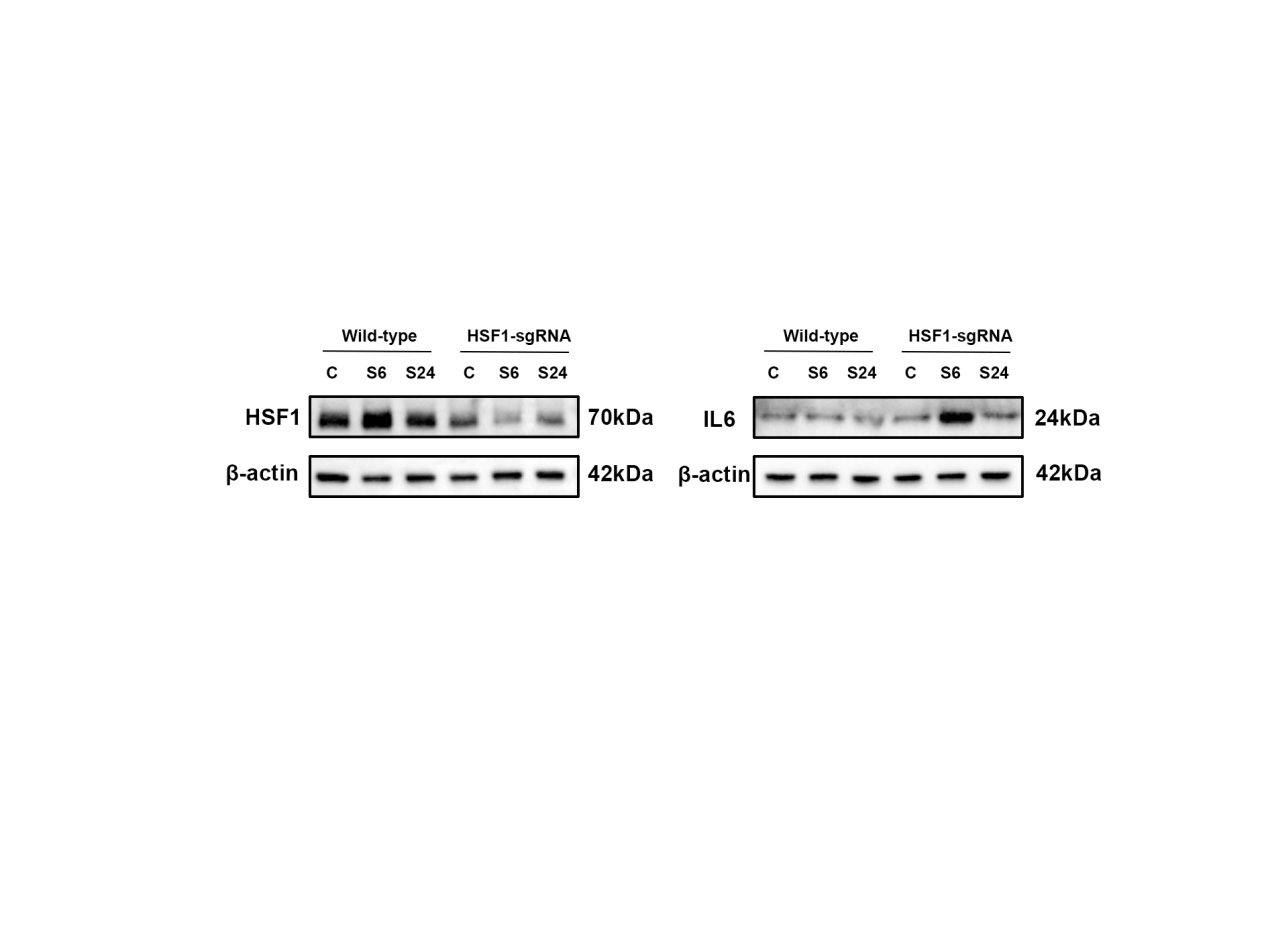


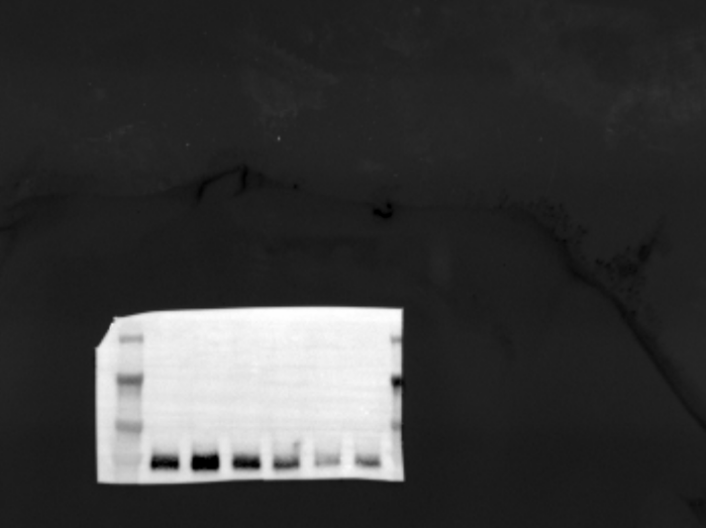
 R-HSF1


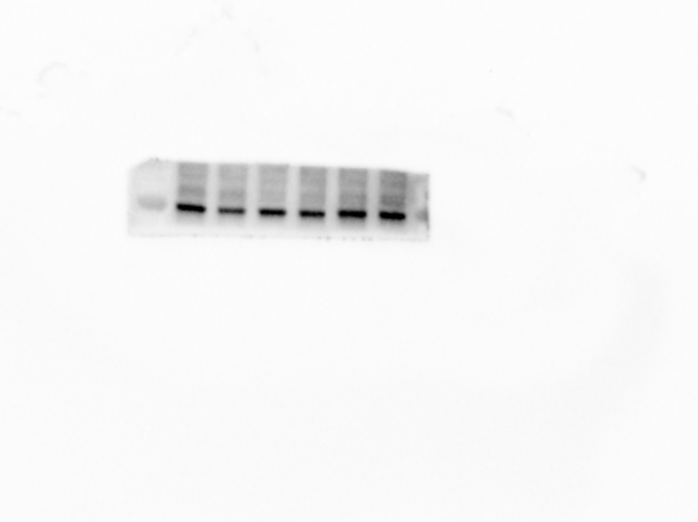
β-actin


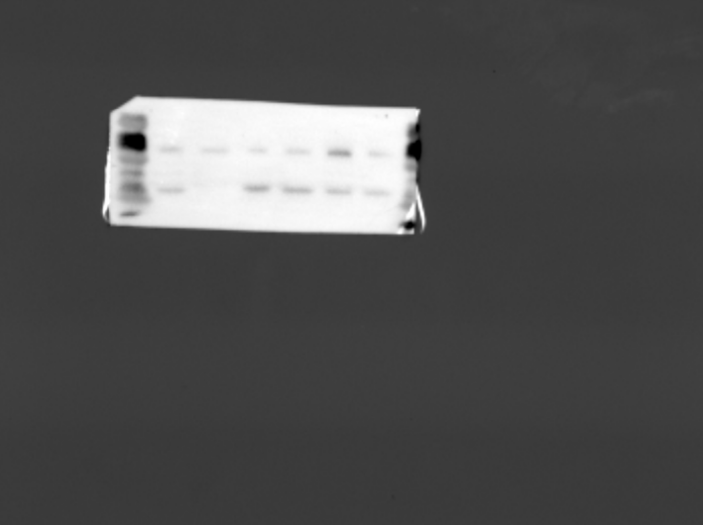
 R-IL6-1


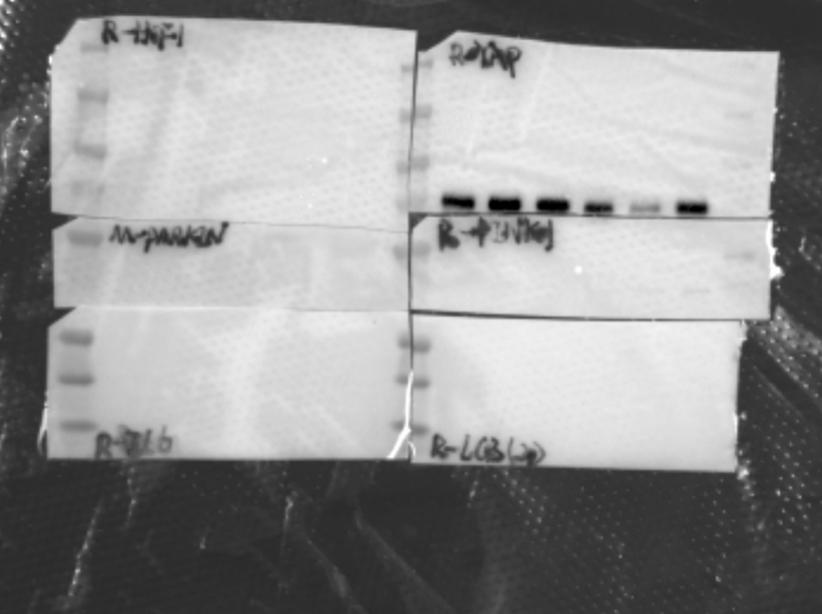
 complete (left side)


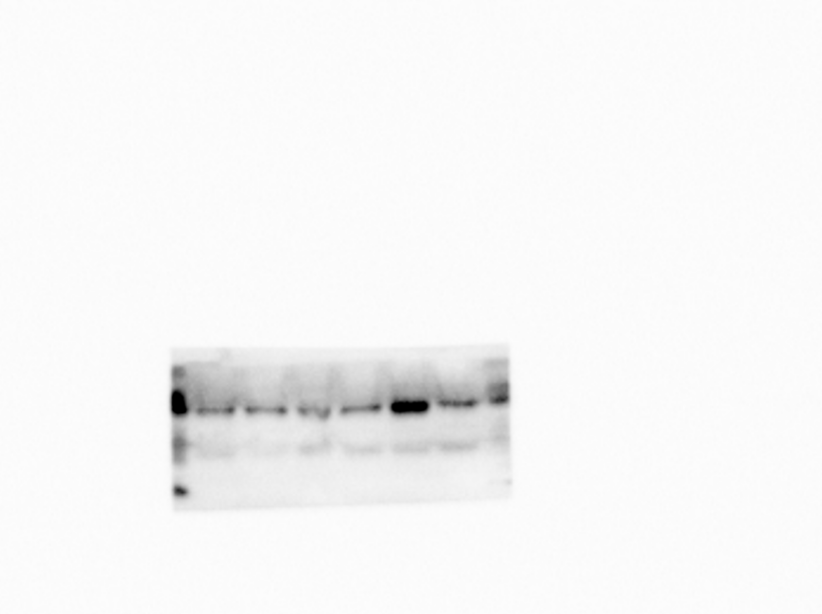
 R-IL6-2


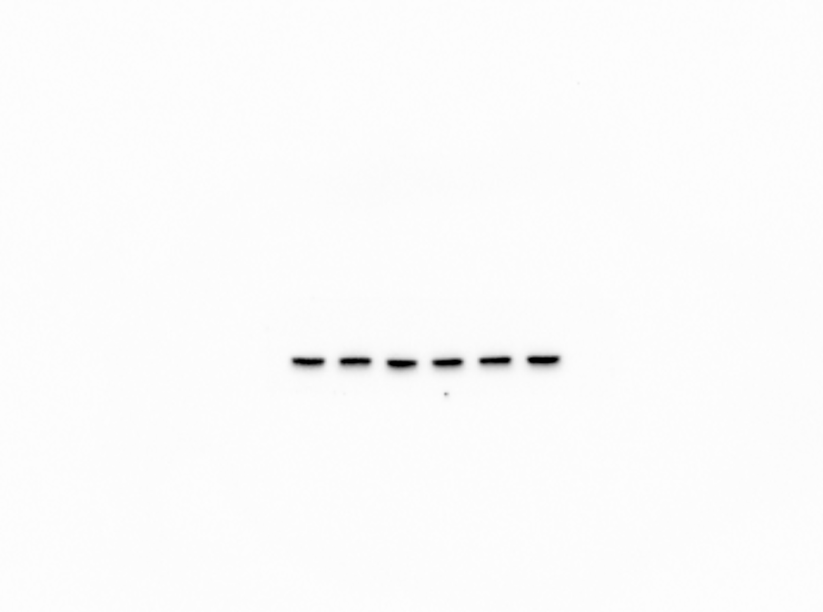
β-actin


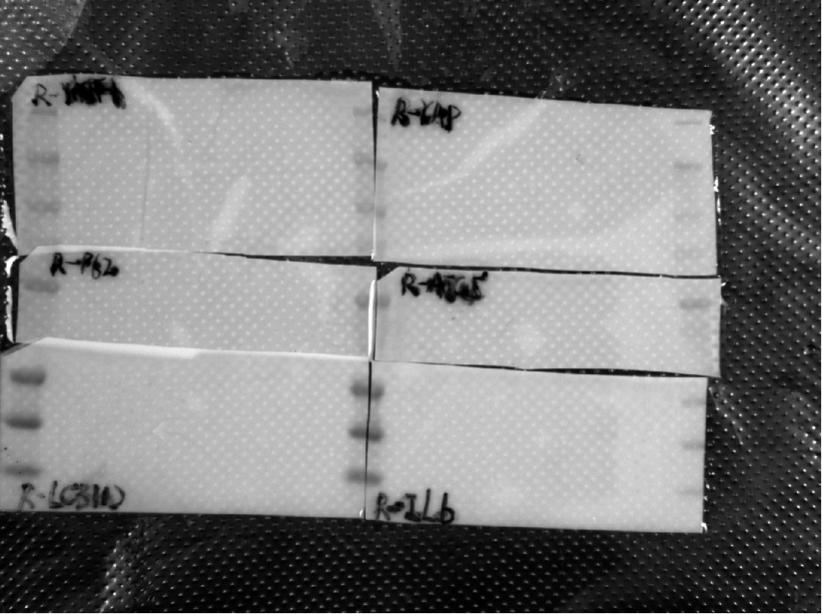
 complete (right side)

Supplement: Supplementary file 2 — Supplementary material [file mmc2.docx]
